# Proximity to Radiotherapy Center, Population, Average Income, and Health Insurance Status as Predictors of Cancer Mortality at the County Level in the United States

**DOI:** 10.1200/GO.23.00130

**Published:** 2023-09-28

**Authors:** Matthew Beckett, Luc Goethals, Ryan D. Kraus, Kseniya Denysenko, Maria Fernanda Barone Mussalem Gentiles, Yaroslav Pynda, May Abdel-Wahab

**Affiliations:** ^1^ The Ottawa Hospital Cancer Centre, Ottawa, Ontario, Canada; ^2^ International Atomic Energy Agency, Vienna, Austria

## Abstract

**PURPOSE:**

Sufficient radiotherapy (RT) capacity is essential to delivery of high-quality cancer care. However, despite sufficient capacity, universal access is not always possible in high-income countries because of factors beyond the commonly used parameter of machines per million people. This study assesses the barriers to RT in a high-income country and how these affect cancer mortality.

**METHODS:**

This cross-sectional study used US county-level data obtained from Center for Disease Control and Prevention and the International Atomic Energy Agency Directory of Radiotherapy Centres. RT facilities in the United States were mapped using Geographic Information Systems software. Univariate analysis was used to identify whether distance to a RT center or various socioeconomic factors were predictive of all-cancer mortality-to-incidence ratio (MIR). Significant variables (*P* ≤ .05) on univariate analysis were included in a step-wise backward elimination method of multiple regression analysis.

**RESULTS:**

Thirty-one percent of US counties have at least one RT facility and 8.3% have five or more. The median linear distance from a county's centroid to the nearest RT center was 36 km, and the median county all-cancer MIR was 0.37. The amount of RT centers, linear accelerators, and brachytherapy units per 1 million people were associated with all-cancer MIR (*P* < .05). Greater distance to RT facilities, lower county population, lower average income per county, and higher proportion of patients without health insurance were associated with increased all-cancer MIR (*R*-squared, 0.2113; *F*, 94.22; *P* < .001).

**CONCLUSION:**

This analysis used unique high-quality data sets to identify significant barriers to RT access that correspond to higher cancer mortality at the county level. Geographic access, personal income, and insurance status all contribute to these concerning disparities. Efforts to address these barriers are needed.

## Teaser

Toward further understanding of the geographic disparities in oncologic outcomes faced by American patients.

## INTRODUCTION

Radiotherapy (RT) is an essential component of evidence-based multidisciplinary cancer care. It is estimated that RT is indicated for approximately 50% of patients with cancer for either curative or palliative treatment.^1-4^ There exists a particularly large role for RT in certain cancers such as those of the head and neck and the uterine cervix, in which localized cancers can be treated definitively with RT.^2^ In high-income settings, when utilized optimally, RT provides significant local control and overall survival benefits at the population level.^4^ However, access to care can be an issue even in high-income countries. Persistent inequities in access to treatment are seen in North America despite sufficient RT capacity for its populations.^5^

**CONTEXT**

**Key Objective**
Radiotherapy (RT) is a vital cornerstone of cancer care. Our objective was to determine how geographic proximity to a RT-equipped cancer center, average income of a county population, and proportion of a county population without health insurance are associated with cancer mortality at the county level in the United States.
**Knowledge Generated**
Counties with geographic centroids further from RT-equipped cancer centers, lower average income, and a higher proportion of patients lacking health insurance had a higher all-cancer mortality-to-incidence ratio.
**Relevance**
Americans face major disparities in oncologic outcomes. Geographic access to RT, personal income, and insurance status all appear to contribute to higher cancer mortality at the county level. By deepening our understanding of these socioeconomic barriers to cancer survival and accounting for them in our patient care, we can work to improve our systems at local and regional levels.

One barrier to access lies in geography. Longer travel distances to radiation centers have been associated with decreased RT utilization, poorer oncologic outcomes, lower patient satisfaction, and higher costs.^6-15^

Socioeconomic status is another barrier. Low socioeconomic status has been associated with fewer consultations to specialist services, lower utilization of RT and other cancer-directed therapies, more treatment delays, less post-treatment follow-up, and poorer survival.^16-22^ Intimately related to socioeconomic status in the United States is the heterogeneous health insurance landscape for patients younger than 65 years. Uninsured and underinsured patients are significantly more likely to present with advanced disease that is less likely to be amenable to curative treatment.^23-25^

Many previous studies have explored the influence of socioeconomic status and insurance on RT usage and outcomes, and several studies have, in specific administrative regions and clinical contexts, investigated the association between proximity to RT center and oncologic outcomes.^7-15,26,27^ Our aim is, therefore, to confirm these findings on a national level by simultaneously exploring the impact of distance to RT center, personal income, and insurance status on cancer mortality using a novel combination of data sets. By deepening our understanding of the issues underlying real-world access to RT in the United States, we hope these findings can inform cancer care policy and administration.

## METHODS

We conducted a cross-sectional study using US county level data on cancer incidence and mortality. There are 3,243 counties, subdivisions of a state, within the United States, each with a unique ID defined by the US Government Census Bureau. All counties in Kansas, MN, and NV, as well as a small number of various counties in other states, did not have cancer incidence and/or mortality data available. Thus, we created a database capturing cancer statistics and demographic data on a county level for 2,824 counties. This information was obtained from the US Census Bureau, the Bureau of Economic Analysis, the Centers for Disease Control and Prevention (CDC), and the International Atomic Energy Agency Directory of Radiotherapy Centres (DIRAC). We obtained a list of US RT centers from DIRAC, an electronic and centralized database of international RT centers created and maintained by the International Atomic Energy Agency.^28^ We then applied Geographic Information Systems (GIS) software (QGIS v.3.16.9) to map all US counties on the basis of data from the Environmental Systems Research Institute.^29^ The geographic center (centroid) of each county was autocalculated. We measured the linear distance from each county centroid to the nearest RT center.

We obtained all-cancer incidence and mortality rates for each county from the CDC over a period of 4 years (2014-2018) on the basis of 2020 submission data. Age-standardized mortality-to-incidence ratios (MIRs) for each county were calculated as the age-standardized mortality rate divided by the age-standardized incidence rate. Data regarding the population of each county and the rate of the population without health insurance was obtained from the US Census Bureau for the year 2019.^30,31^ We also obtained data on personal income from each county from the US Bureau of Economic Analysis.^32^

We performed a univariate analysis to identify variables that predicted all-cancer MIR. Independent variables in this analysis included distance to RT center, county population, mean county personal income, and proportion of the county population without health insurance. Variables that were deemed to be significant (*P* ≤ .05) on univariate analysis were then included in a step-wise backward elimination method of multiple regression analysis (ordinary least squares). Variables were dropped if found to have high multicollinearity within the model as indicated by a variance inflation score of >5 and then in order of least significance, until all remaining variables were significantly associated at *P* ≤ .05.

## RESULTS

**FIG 1:**
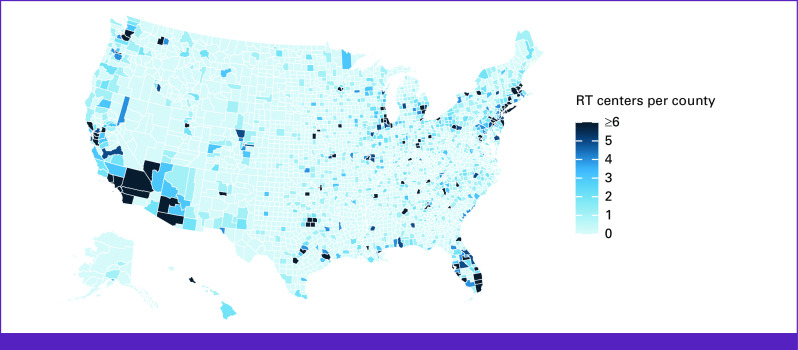
Map of RT facilities by county. Counties are illustrated in darkening shades of blue corresponding to higher number of RT centers within the county boundaries. RT, radiotherapy.

**FIG 2:**
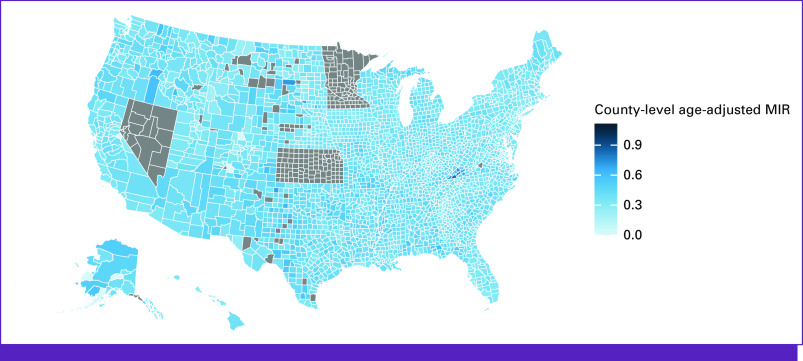
Map of age-adjusted MIR by county. Counties are illustrated in darkening shades of blue corresponding to higher age adjusted MIRs. Counties in gray did not have data available for analysis. Gray indicates counties for which data were not available. MIR, mortality-to-incidence ratio.

Of all counties included in our study, 69% did not have a RT facility therein while 8.3% of counties were found to contain five or more RT facilities. The median linear distance from county centroid to nearest RT center was 36 km. The closest distance was in Newberry County, SC (0.053 km) and the furthest distance in Petersburg Borough, AK (1,176.5 km). A map of RT facilities is illustrated in Figure 1. For the counties included in our study, the median all-cancer MIR was 0.37. The lowest all-cancer MIR was in Greeley County, NE (0.14), and the highest all-cancer MIR was in Scott County, VA (0.86). County MIRs are illustrated in Figure 2.

Descriptive statistics for each independent variable considered in the regression analysis for all-cancer MIR are presented in Table 1. In univariate analysis, distance to RT and each sociodemographic variable were significantly associated with all-cancer MIR (*P* < .05).

**TABLE 1:**
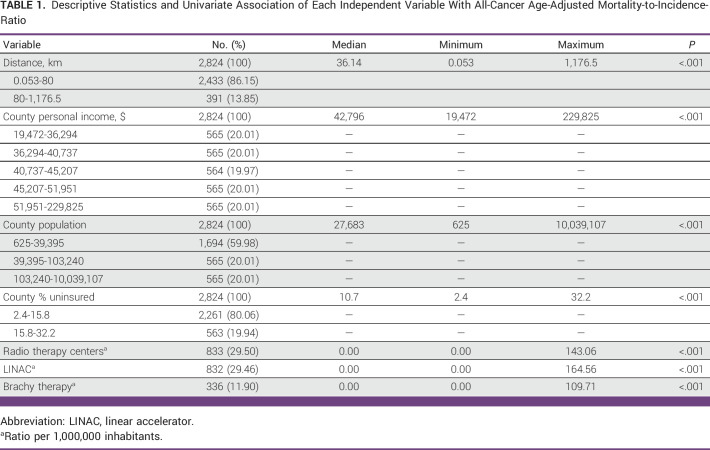
Descriptive Statistics and Univariate Association of Each Independent Variable With All-Cancer Age-Adjusted Mortality-to-Incidence-Ratio

The median county population size was 27,683 in 2019. The number of RT centers, linear accelerators, and brachytherapy units per 1 million people were significantly associated with all-cancer MIR (*P* < .05). In the regression model for all-cancer MIR, greater distance to RT facilities, lower county population, lower average income per county, and higher proportion of patients without health insurance were statistically significant predictors of increased all-cancer MIR (*R*-squared, 0.2113; *F*, 94.22; *P* < .001), as shown in Table 2.

**TABLE 2:**
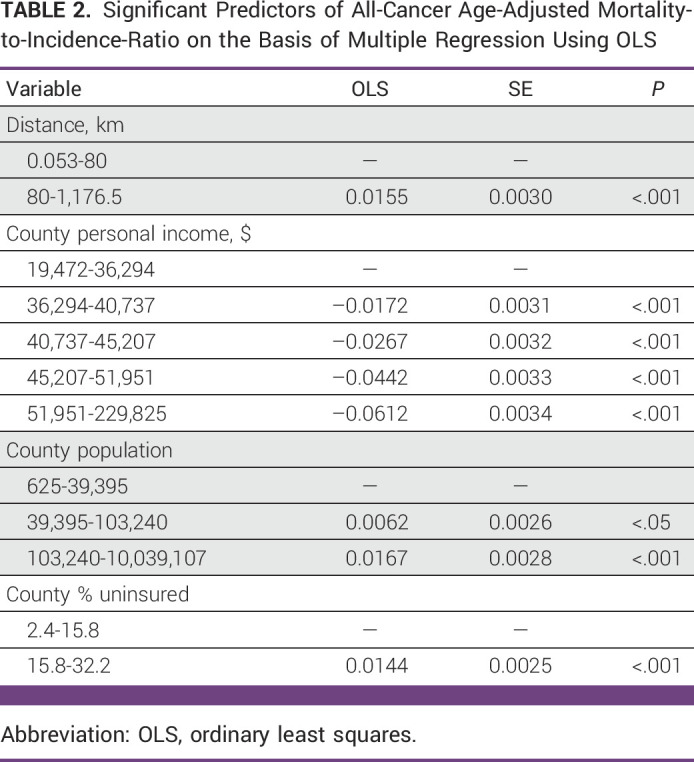
Significant Predictors of All-Cancer Age-Adjusted Mortality-to-Incidence-Ratio on the Basis of Multiple Regression Using OLS

## DISCUSSION

Our study provides further evidence that geographic access, income, and health insurance status significantly affect oncologic outcomes. Addressing the disparities of oncologic outcomes between patients of differing socioeconomic status should be one of the highest priorities in the United States as an estimated 22% of cancer deaths in the United States could be avoided if these disparities did not exist.^33,34^ Improving our understanding of these issues at the population level will allow us to properly contextualize potential solutions.

The United States has populations largely concentrated on the East and West Coasts and a more sparsely populated central region. This population distribution influences patient access to major cancer centers, leaving many patients with cancer with no choice but to travel long distances to receive high-quality cancer care. Unfortunately, our study found that the distance a patient must travel to receive appropriate cancer care, such as RT, has been associated higher overall cancer mortality rates.

The relationship between increased travel distance to a RT center and higher MIRs is likely multifactorial. For patients living further from RT centers, RT may necessitate hours of driving to and from hospital for several weeks; this travel burden may simply be a prohibitive barrier to treatment. It is, therefore, unsurprising that patients facing long travel distances are less likely to receive RT.^6,10^ Distance from a RT center has been found to affect which treatments patients pursue, with patients living further from RT facilities more commonly choosing surgical interventions, or more aggressive surgical procedures, to avoid RT.^6,11,13^ Patients who require RT after surgical interventions such as lumpectomies are half as likely to receive RT if they live 75 miles or more from a RT facility, a deviation from guideline-recommended standard of care.^6^ Deviations from standards of care such as these may in part explain our findings of an association between distance to RT facilities and higher MIR. In addition to delivery of RT, specialized services at RT-equipped cancer centers include cancer screening, diagnostic workups, and monitoring of patients for recurrent disease. These are essential services for patients with cancer, and patients who must travel longer distances to receive these services have also experienced delays in diagnosis and therefore present with more advanced stages of disease, further compounding the effects of non–standard-of-care treatments and worsening MIRs among these patient populations.^10^

This maldistribution of RT facilities may be due to a lack of centralized leadership on a national or regional level. Geospatial mapping may assist in determining optimal location for RT facilities on the basis of areas with elevated MIR, as identified in our study. The infrastructure required to deliver RT is costly, and RT facilities established in areas without sufficient patient volume are unlikely to be sustainable without external financial support. Alternative approaches to decreasing travel burden may require changes to practice patterns, such as the increased utilization of hypofractionated treatments to shorten the overall duration of treatment by delivering fewer treatments with higher daily radiation doses. Hypofractionated treatments have become the standard of care for patients with breast and prostate cancer as they provide noninferior oncologic outcomes and have a similar toxicity profile.^35-41^ After completion of RT, patients may be able to receive follow-up care at radiation oncology satellite clinics in rural communities, limiting the amount of travel required for nontreatment visits. The COVID-19 era has also resulted in the rise of telemedicine, another tool to help minimize travel for nontreatment visits.

The challenges faced by patients in rural and remote areas are clear. However, urban-dwelling patients also face barriers in access to treatment, as 23% of counties with inadequate coverage by major cancer centers have been classified as being in the highest population density quintile of the United States.^42^ This highlights the fact that lack of access to high-quality cancer care is an issue for both rural and urban American populations, as geographic proximity to a RT center does not guarantee equitable access to RT. Contributing factors to these disparities include limited financial resources and health insurance status.

With respect to financial resources, we found that counties with lower average incomes had less favorable MIRs. This is consistent with previous reports that lower-income patients have higher mortality rates than more affluent patients.^34,43,44^ A lack of financial resources may affect access to RT in a number of ways. For rural patients, transportation or housing near the treatment center may be prohibitively costly. Patients in urban settings may live in closer proximity to a RT center, but, due to financial constraints, may need to take lengthy public transportation routes to reach a treatment center, mirroring the challenges faced by their rural counterparts. Travel costs are not insignificant; studies have reported that travel expenses for a course of RT can frequently amount to more than $1,500 with the cost increasing the further a patient lives from a treatment center, even in urban settings.^45,46^

Low-income patients also face challenges related to employment during and after treatment. Approximately 45% of patients with cancer are between the age 20 and 64 years and are working full-time or part-time.^47^ Low-income workers are less likely to work in accommodating environments that allow paid sick leave, flexible schedules, or flexibility in tasks, all of which are crucial for a patient undergoing cancer treatment.^48^ Patients who had reduced ability to work during treatment and those with less support from their employers during treatment were more likely to require long-term sick leave away from work once treatment had completed.^49^ These issues may also extend to a patient's support network and caregivers.^49^ A patient's care needs during a course of RT can, in some cases, be life-altering, necessitating a strong and flexible support network at work and home in order for a patient to successfully complete treatment.

As above, hypofractionation may provide a solution. In Canada, decreasing the duration of RT for prostate cancer from 39 fractions to 5 fractions was found to reduce out-of-pocket expenses for patients by C$1,930 when accounting for traveling expenses alone.^46^ Similar results were seen in the United States, where the cost to the patient in nonmedical expenses was approximately 50% less when using a 15-fraction treatment approach compared with a 25-fraction treatment approach for breast cancer.^45^ These hypofractionated treatment regimens have also been found to improve treatment compliance and the rates of treatment completion.^50-52^

Specialized financial navigators may be able to assist patients in access to medications or copay or premium assistance, insurance enrollment, and housing or transportation programs. Hospitals that have implemented Financial Navigator Programs decreased patients' out-of-pocket expenditures while increasing hospital revenue by connecting patients with sources of funding that otherwise would have been provided as charity care.^53^ This increased hospital revenue should be used to address related barriers to care by providing transportation and lodging for patients while on treatment and survivorship care. These cancer lodges have been shown to reduce out-of-pocket patient costs by up to 80%.^54^ Efforts should also be made to minimize hospital-controlled patient expenses such as parking fees. As of 2020, 32% of NCI-designated cancer centers charged patients for parking during their radiation appointments.^55^ These seemingly minor expenses can accumulate to become a large financial burden for patients with cancer.

After diagnosis, uninsured patients and those with Medicaid or Medicare are less likely to undergo high-quality treatment such as RT or survivorship care to monitor for disease recurrence.^56-58^ They are more likely to receive treatment at rural, urban nonteaching, private investor-owned, or government (nonfederal) hospitals, which are associated with inferior survival outcomes compared with urban teaching hospitals.^59^ For these reasons, it is not surprising that our study identified higher MIRs in counties with large uninsured populations consistent with previously published reports.^60^

The United States has a heterogeneous health insurance landscape with most patients receiving health insurance through their employer, followed by Medicaid (20%), Medicare (18%), and a significant proportion of patients being uninsured (9%) on the basis of 2019 data.^61^ For patients with cancer, health insurance has long represented one of the most significant determinants of access to care.^62^ This is illustrated through multiple reports which have found that uninsured patients are less likely to receive preventative medical interventions to decrease the risk of developing cancer, participate in age-appropriate cancer screening, and receive appropriate follow-up for abnormal screening results. These patients are, therefore, more likely to present with cancer of more advanced stages that are less amenable to curative treatments.^12-15,24,59,60^

Potential interventions differ in scope and feasibility but range from implementing universal health insurance to creating a national paid leave policy for patients with cancer.^63^ Low barrier interventions might include expanding Medicaid eligibility further in additional states.^64^ The United States has previously had success in leveraging health care policy to improve cancer outcomes. This was most recently accomplished through legislation that improved access to cancer care by decreasing rates of patients being uninsured and decreasing cost sharing for preventative services.^64,65^ Dependent coverage expansion for age 19-25 years has been shown to lower rates of the uninsured and lead to more preventative health interventions, diagnosis at earlier stages of disease, and more timely receipt of definitive cancer treatment.^66,67^ Additionally, patients diagnosed with cancer who lived in states that expanded Medicaid eligibility were more likely to be insured, present with an earlier stage of disease, and have access to treatment compared with patients living in states that did not expand Medicaid eligibility.^64,66^ In these states with expanded Medicaid eligibility, historic disparities in the insurance status of low-income, minority, and rural patients with cancer decreased.^64^

Change in deductible policy may also benefit underinsured patients. Deductibles are the required amount paid by the patient before health insurance coverage starts covering medical bills. Patients with cancer enrolled in high deductible plans are more likely to delay or forgo essential medical care because of the associated financial toxicity compared with patients with low-deductible plans.^68-70^ One solution may be through the use of value-based insurance design, where essential services such as cancer screening would not have an associated deductible.^65^ These value-based insurance designs have been shown to decrease out-of-pocket expenses, improve access to high-value services, and reduce health care disparities.^71^

Population-level analysis bears inherent limitations. We are not privy to details of individual patients and how the complex array of factors discussed above, and others, might intersect to inform treatment decisions and influence oncologic outcomes in a specific case. Our analysis is based on county-level data, but disparities may occur on a more granular level, differing between neighborhoods, communities, and groups that overlap geographically and cannot be captured with these methods. The dimension of race and ethnicity were not investigated. Furthermore, the centroid of a county was used as a standardized way to assess a county's geographic proximity to a RT center, but this point is, again, artificial and may not be a meaningful point for every county's population as the centroid of a county might not correlate with the area of highest population density. Additionally, where a patient with cancer can receive RT treatment is, in part, dictated by their insurance coverage rather than being solely on the basis of geographic proximity. Many patients may forgo treatment at the closest RT facilities to receive treatment at an in-network facility posing challenges in interpreting the association between oncologic outcomes and proximity to RT facilities.

Further variables regarding RT capabilities at the identified cancer centers, such as staffing and days of utilization, may also be relevant. Such information was not available at the scale which would have been necessary to integrate these variables into this study but might be valuable in more focused future investigations.

RT is not indicated for every patient with cancer. However, MIRs used in this study are based on all cancers. Future investigations might seek to use similar methods to assess outcomes of particular cancers, such as head and neck or cervical cancers, for which RT is more consistently indicated. However, it should be noted that, as some counties have very small populations, some data are likely to be censored in administrative databases to protect patient confidentiality, and MIR may not be a functional metric with smaller numbers.

Despite these limitations, this study has improved our understanding of barriers faced by American patients with cancer in their pursuit of high-quality cancer care on a systemic level. Furthermore, comparisons of geographic access to RT, systemic therapy, and cancer-directed surgery may draw attention to barriers and solutions specific to various treatment modalities.

In conclusion, as we strive to individualize cancer treatment for every patient, developing guidance on the basis of molecular marker, histological subtype, and degree of tumor burden, a patient's access to these advances is dependent on many external factors. We must seek to understand how these complexities inform the way patients navigate the medical system. Our analysis has used unique, high-quality data sets to identify significant barriers to RT access that correspond to higher cancer mortality at the county level. Geographic access, personal income, and insurance status all contribute to these concerning disparities. By deepening our understanding of these socioeconomic barriers to cancer survival and accounting for them in our patient care, we can work to improve our systems at local and regional levels and alleviate burden of cancer on individuals, families, and communities.
